# The cricketer’s shoulder and injury: Asymmetries in range of movement and muscle length

**DOI:** 10.4102/sajp.v76i1.754

**Published:** 2020-03-11

**Authors:** Benita Olivier, Bhakti Lala, Nadia Gillion

**Affiliations:** 1 Department of Physiotherapy, School of Therapeutic Sciences, Faculty of Health Sciences, University of the Witwatersrand, Johannesburg, South Africa

**Keywords:** shoulder injuries, sports, cricket, range of movement, throwing arc, flexibility

## Abstract

**Background:**

Shoulder injuries in cricket are often undetected and untreated.

**Objectives:**

To determine whether there are associations between shoulder internal and external rotation range of movement (ROM), throwing arc (TA) ROM, glenohumeral internal rotation deficit (GIRD), external rotation gain (ERG), pectoralis minor muscle length and the incidence of shoulder injury during the first 3 months of a cricket season amongst provincial and club cricketers.

**Method:**

Male, actively participating, provincial and club cricketers were included in this prospective longitudinal cohort study. The independent variables included shoulder pain, which did not limit participation in cricket training and matches; shoulder external and internal rotation (ROM, TA ROM, GIRD and ERG) and pectoralis minor muscle length. Time-loss dominant shoulder injury was recorded for 3 months.

**Results:**

Nine of the 32 participants sustained dominant shoulder injuries. Initial non-time-loss shoulder pain during baseline testing was associated with time-loss in-season shoulder injury (*p* = 0.007). Statistically significant side-to-side differences were found for all of the independent variables (internal rotation ROM, TA ROM and pectoralis minor muscle length distance), with the exception of external rotation ROM, amongst the uninjured players.

**Conclusion:**

Non-time-loss-defined shoulder pain in actively participating cricketers seems to be a precursor to time-loss shoulder injury. Asymmetries in ROM and pectoralis minor muscle length in uninjured cricketers may have a protective role to play in the case of shoulder injury.

**Clinical implications:**

The presence of shoulder pain and asymmetries in ROM should be investigated during the pre-season screening procedures, and early intervention should be implemented where appropriate.

## Introduction

The prevalence rate of seasonal shoulder injury in professional cricketers is 23%, which includes player-reported injuries (i.e. pain experienced during cricket matches, training and activities of daily living) (Ranson & Gregory 2008). After lower back, thigh and knee injuries, the shoulder is the body area with the fourth-highest incidence with respect to non-time-loss injury amongst cricketers who participated in the International Cricket Council (ICC) Cricket World Cup 2011 (Ranson et al. 2013).

Repeated end of range movement may influence the shoulder structures. Glenohumeral joint injuries in overhead athletes occur mostly during the late cocking (Mihata et al. 2015) and the follow-through phase of throwing (Kinsella et al. 2014). With repetitive throwing, the anterior inferior glenohumeral ligament stretches during the late cocking phase (Mihata et al. 2015), or humeral retroversion adaptations occur (Reagan et al. 2002), resulting in an increase in the external rotation (ER) range of movement (ROM) and an anterior capsule instability (Mihata et al. 2015).

During the deceleration and follow-through phase, excessive distractive forces may cause tearing of, or tension in, the posterior joint capsule, and subsequent healing may result in a decline in internal rotation (IR) ROM (Dashottar & Borstad 2012).

Side-to-side differences in shoulder ROM may predict injury during the cricket season. Cricketers experiencing a gradual onset of shoulder pain present with an IR side-to-side difference of 10.0°, whilst those without pain present with an IR difference of 6.8° (*p* = 0.03) (Giles & Musa 2008). Reduced glenohumeral IR on the dominant side, as opposed to the non-dominant side, was present in both groups (Giles & Musa 2008). In elite female cricket fast bowlers, IR ROM was reduced in the bowling shoulder as opposed to the non-bowling shoulder amongst bowlers with a history of shoulder pain (Stuelcken, Ginn & Sinclair 2008). This side-to-side difference was not present in the pain-free group. However, in terms of ER, an ER side-to-side difference of less than 5° may be a predictor of future shoulder injury (Manske et al. 2013). Glenohumeral internal rotation deficit (GIRD) and external rotation gain (ERG) have been identified in cricketers (Giles & Musa 2008) as contributors to shoulder injuries. Throwing arc (TA) ROM and its association with shoulder injury has been studied in an elite female fast-bowling population (Stuelcken et al. 2008), and in a mixed female and male population consisting of adolescent and adult elite cricketers (Giles & Musa 2008). Throwing arc ROM has not been studied in a male-only population of cricketers older than 18 years.

A shortened pectoralis minor muscle changes the kinematics of the scapula, which could contribute to a predisposition to shoulder injury (Borstad & Ludewig 2005). This abnormal scapula positioning brings the glenoid cavity and the greater tuberosity closer together, which impinges on the posterior shoulder tissue during the cocking phase of throwing (Kinsella et al. 2014). The contribution of pectoralis minor muscle length to shoulder injury in cricketers remains unclear.

Shoulder rotation ROM, as well as pectoralis minor muscle length, may play a role in time-loss shoulder injury in cricketers. The determination of shoulder rotation ROM and pectoralis muscle length and its potential association with shoulder injury in cricketers may inform the development of shoulder injury screening tools. Early identification of injury risk could contribute to more effective injury prevention measures and reduce time off from participation. Therefore, the aim of this study was to determine whether there are associations between pre-season shoulder IR and ER ROM, TA ROM, GIRD, ERG and pectoralis minor muscle length and the incidence of reported shoulder injury during the first 3 months of a cricket season amongst provincial and club cricketers.

## Methods

This prospective longitudinal cohort study included 36 elite male cricketers who volunteered to participate in the study. Post hoc power calculations were run using G*Power (3.1) on four of the outcomes, the average power of which was 0.81.

Participants were included if they were male, provincial or premier league club cricketers, aged 18 years or older and exposed to a weekly cricket training workload. Players from the included teams were exposed to similar pre-season training routines, in-season training sessions and similar game schedules, that is, all the players participated in training sessions as well as multiple games weekly, and all of the teams involved in the study participated in the same T20 league. As it is common for athletes with musculoskeletal complaints to actively participate in sports (Ranson & Gregory 2008), players who were experiencing dominant shoulder pain at the time of data collection but who were actively participating (i.e. who made themselves available for cricket activities and who were thus not excluded on the basis of the injury definition used in this study) were also included. Players were excluded if they had previous orthopaedic-related surgery, had not been exposed to a weekly cricket training workload, had a heart condition or who were currently using anti-inflammatories or analgesics.

### Definition of injury

An injury was defined as any player-reported non-contact, dominant shoulder pain and/or injury that occurs in a player during or after cricket training or a match involving batting, bowling, fielding or wicket keeping, and resulting in the cessation or limitation of that sporting activity (Orchard et al. 2016).

### Procedures

#### Pilot study

Between-day intra-rater reliability of all measurements was determined during the pilot study and involved four participants. Inter-rater reliability was not determined because all measurements were performed by the second author (B.L.) during the main study. The intra-rater reliability was determined for dominant (*r* = 1.00; *p* = 0.01) and non-dominant (*r* = 0.32; *p* = 0.68) IR, and dominant (*r* = 1.00; *p* = 0.01) and non-dominant (*r* = 1.00; *p* = 0.01) ER ROM, using a manual inclinometer, as well as for dominant (*r* = 0.83; *p* = 0.17) and non-dominant (*r* = 0.80; *p* = 0.20) ‘pectoralis minor muscle length test distance’ using a right-angle ruler.

#### Main study

For the main study, participants were measured pre-season and during the first 3 weeks of the in-season.

To measure ER and IR, with the participant lying in supine, a research assistant stabilised the scapula by applying pressure over the coracoid process for shoulder rotation ROM measurements. The second author (B.L.) passively abducted the shoulder into a 90° abduction position and full passive ER or IR, with the manual inclinometer (Kolber & Hanney 2012) placed on the anterior distal forearm for ER and the posterior distal forearm for IR (Sundaram, Skn & Karuppannan 2012). Each measurement was performed twice and the average was calculated.

The pectoralis minor muscle length was assessed through the ‘pectoralis minor muscle length test distance’ (acromion to plinth distance) (Lewis & Valentine 2007). The participant rested in supine on the plinth, elbows flexed and hands resting on the lateral part of the abdomen. Using a rigid plastic right-angled ruler (Lewis & Valentine 2007), the second author (B.L.) measured the distance from the posterior part of the acromion process to the plinth in centimetres on both shoulders.

The incidence of injuries was monitored for 3 months using a standard injury report form enquiring on type, nature and mechanism of injury. It is worth noting that the first 3 months of the season involved a high workload period for training and matches for all of the teams involved in our study. Participants were contacted weekly, either telephonically or in person, regarding their dominant shoulder injury status and for the authors to obtain information relating to their training and match workloads. Injuries were reported to the first author, team physiotherapist, coach or trainer.

### Data reduction

The following calculations were performed using the data collected, namely, IR and ER ROM (Almeida et al. 2013): GIRD = non-dominant minus dominant shoulder IR ROM; ERG = non-dominant minus dominant ER ROM; and TA ROM = shoulder IR ROM plus shoulder ER ROM (also called the total rotation motion – TRM).

### Statistical analysis

Data were analysed using International Business Machines (IBM) Statistical Package for the Social Sciences (SPSS) Statistics 25. Spearman’s correlation test was used to analyse the intra-rater reliability of the shoulder IR and ER ROM, TA ROM and ‘pectoralis minor muscle length test distance’. The correlation was evaluated as minimal or no relationship present (0.00 < *r* < 0.25), fair (0.25 < *r* < 0.50), moderate (0.50 < *r* < 0.75) or excellent (*r* > 0.75) (Portney & Watkins 2009).

Non-parametric tests were applied because of the small sample size. Descriptive data are presented as medians and inter-quartile ranges (IQRs) (25–75th percentile). The Mann–Whitney U test was used to compare variables between the injured and uninjured groups. The Wilcoxon Signed Ranked test was used to compare variables on the dominant to the non-dominant sides. Effect sizes were calculated using the *Z* score in the formula *r* = z/√*n* (Pautz et al. 2018a). Effect sizes of 0.2, 0.5 and 0.8 were interpreted as small, medium and large, respectively (Pautz et al. 2018a, 2018b). The association between shoulder pain at baseline and in-season injury was determined using a Fisher exact test, with φ to show the effect size.

### Ethical considerations

Ethical clearance was obtained from the University of the Witwatersrand’s human research ethics committee (reference number: M150612). Permission was obtained from the provincial cricket board and cricket club management to invite the team players. Participants provided voluntary written consent and were allowed to withdraw from the study at any time without suffering any repercussions.

## Results

### Participants

Shoulder measurements were completed for 36 male provincial (*n* = 30) and club (*n* = 6) cricketers. The data pertaining to four participants were removed before the analysis, as they had not been exposed to the typical cricket training and match workloads during the season.

The baseline data for 32 participants with a mean age of 23.56 years (SD ± 4.27; range 18–33) were analysed.

Nine (28%) of the 32 participants with a mean age of 23.0 years (SD ± 5.26) sustained dominant shoulder injuries, resulting in pain, which limited their participation, whilst 23 participants with a mean age of 23.78 years (SD ± 3.93) remained uninjured. The mean age was similar for the injured and the uninjured groups (*p* = 0.65). All of the shoulder injuries were on the dominant side and were examples of the gradual onset of non-contact injuries, eight of which occurred as a result of throwing, whilst one was as a result of bowling.

### The relationship between initial pain and injury

Six of the nine participants who sustained a dominant time-loss shoulder injury during the season complained of shoulder pain (which did not limit their participation) during baseline testing. On the other hand, of those who remained injury-free (*n* = 23), only four experienced shoulder pain during baseline testing.

A Fisher’s exact test found an association between injury classification and initial pain (*p* = 0.013), with a moderate effect size (φ = 0.478; *p* = 0.007).

### The difference between the injured and uninjured participants

There were no differences in pre-season measurement of any of the ROM-related variables between the injured and the uninjured groups (Table 1).

**TABLE 1:** A comparison between the injured and uninjured group.

Variable	Injured (*n* = 9)		Uninjured (*n* = 23)		Effect size	*p*
	Median	IQR	Median	25–75th		
Dominant IR ROM (°)	50.0	45–58	50.0	38–64	0.033	0.869
Dominant ER ROM (°)	124.0	110–134	122.0	112–130	0.011	0.967
Dominant TA ROM (°)	180.0	160–186	172.0	160–188	0.026	0.902
Dominant pectoralis minor muscle length test distance (cm)	5.1	4.3–5.95	5.8	5.2–6.4	0.205	0.112
Non-dominant IR ROM (°)	60.0	45–71	62.0	56–76	0.126	0.483
Non-dominant ER ROM (°)	114.0	107–132	120.0	110–130	0.070	0.711
Non-dominant TA ROM (°)	178.0	159–198	182.0	168–200	0.130	0.483
Non-dominant pectoralis minor muscle length test distance (cm)	5.2	3.85–5.8	4.6	4.0–5.4	0.074	0.681
ERG (°)†	0.0	−17–9	−2.0	−6–4	0.040	0.837
GIRD (°)†	10.0	0–18	14.0	0–24	0.119	0.509

IR, internal rotation; ER, external rotation; TA, throwing arc; ERG, external rotation gain; GIRD, glenohumeral internal rotation deficit; IQR, interquartile range (25th–75th percentile); ROM, range of movement.

† , A negative value occurs when the non-dominant rotation ROM is smaller than the dominant rotation ROM.

### The difference between the dominant and non-dominant sides

Side-to-side differences were found amongst the uninjured players in most of the independent variables (IR ROM, TA ROM and ‘pectoralis minor muscle length distance’), with the exception of ER ROM (Table 2).

**TABLE 2:** A comparison between the dominant and non-dominant shoulder in the entire group, injured and uninjured group.

Group	Variable	Dominant		Non-dominant		Effect size	*p*
		Median	IQR	Median	IQR		
Entire group (*n* = 32)	IR ROM (°)	50.00	40.50–62	61.00	53–73.50	0.66	< 0.0001
	ER ROM (°)	123.00	112–130	117.00	110–130	0.15	0.3900
	TA ROM (°)	173.00	160–186	181.00	168–197.50	0.49	0.0100
	Pectoralis minor muscle length test distance (cm)	5.60	5–6.18	4.65	4.05–5.68	0.63	0.0001
Injured group (*n* = 9)	IR ROM (°)	50.00	45–58	60.00	45–71	0.54	0.1100
	ER ROM (°)	124.00	110–134	114.00	107–132	0.23	0.4800
	TA ROM (°)	180.00	160–186	178.00	159–198	0.38	0.2600
	Pectoralis minor muscle length test distance (cm)	5.10	4.30–5.95	5.20	3.85–5.80	0.42	0.2100
Uninjured group (*n* = 23)	IR ROM (°)	50.00	38–64	62.00	56–76	0.69	0.0010
	ER ROM (°)	122.00	112–130	120.00	110–130	0.15	0.4600
	TA ROM (°)	172.00	160–188	182.00	168–200	0.57	0.0100
	Pectoralis minor muscle length test distance (cm)	5.80	5.2–6.4	4.60	4–5.4	0.70	0.0010

IR, internal rotation; ER, external rotation; TA, throwing arc; ERG, external rotation gain; IQR, interquartile range (25th–75th percentile); ROM, range of movement.

In the uninjured group, the ‘pectoralis minor muscle length test distance’ on the dominant side (median = 5.8 cm; IQR = 5.2 cm – 6.4 cm) was larger than that on the non-dominant side (median = 4.6 cm; IQR = 4 cm – 5.4 cm). Thus, the muscle on the dominant side was shorter (*p* = 0.001). This finding presented with a large effect size (*r* = 0.70; *p* = 0.001).

Amongst those who sustained a time-loss injury during the season (injured group), a medium effect size (*r* = 0.54) was found where the dominant IR ROM (median = 50°; IQR = 45° – 58°) is less than the non-dominant IR ROM (median = 60°; IQR = 45° – 71°; *p* = 0.11) (Table 2). A similar finding but with a stronger effect size (*r* = 0.69) was found in the uninjured group (dominant median = 50°; IQR = 38° – 64°; non-dominant = 62°; IQR = 56° – 76°; *p* = 0.001).

## Discussion

As opposed to four of the 23 uninjured participants, six of the nine cricketers who suffered time-loss shoulder injuries on the dominant side reported initial non-time-loss shoulder pain during baseline testing. In our study, and amongst cricketers from England and Wales (Giles & Musa 2008; Ranson & Gregory 2008), players with shoulder pain continued to train and play games, thus affecting their performance, especially in terms of their bowling and fielding capabilities (Ranson & Gregory 2008). As shown by our results, players with injuries not yet causing missed playing time need to be flagged because such injuries might lead to missed playing time in the future. Also, because of the importance of identifying such injuries early on, a definition for ‘medical attention injuries’ was added to the international consensus statement on injury surveillance in cricket (Orchard et al. 2016).

No differences in IR ROM, ER ROM, GIRD or ERG were noted between the injured and uninjured players.

In fact, similar findings were present in other studies where no IR ROM or ER ROM differences between injured and uninjured players were found in elite female fast bowlers (Stuelcken et al. 2008) and male provincial fast bowlers (Aginsky, Lategan & Stretch 2004). The lack of differentiation between the injured and uninjured players in terms of flexibility measures, but with consideration being given to the presence of side-to-side differences, leads to the deduction that flexibility measurements should be assessed on an individual basis and that the measurements for each athlete should be compared to the earlier ones pertaining to the person in question, rather than to those of a different athlete.

A side-to-side difference in IR ROM (12.00°) was evident in the uninjured group, with reduced IR ROM on the dominant shoulder as opposed to the non-dominant shoulder. It should be noted that a similar side-to-side difference was present amongst the injured group, although the effect size was weaker than what was found in the uninjured group. Limb ROM asymmetries were identified in injury-free spin bowlers (12.90°) and fast bowlers (11.58°) (Sundaram et al. 2012). These side-to-side differences may occur as a result of the repetitive nature of throwing and bowling, which causes soft tissue adaptations and the resultant contracture of the posterior glenohumeral joint capsule, resulting in a decreased IR ROM (Dashottar & Borstad 2012).

When comparisons in terms of ER ROM were made in the injured and uninjured subgroups, no side-to-side differences were found. On the contrary, significant ER ROM asymmetry, with more ER ROM, in the bowling shoulder, was identified in uninjured female fast bowlers (Stuelcken et al. 2008). Amongst cricketers with a history of a gradual onset of shoulder pain, more symmetry was shown in terms of ER ROM than was the case in the no-pain group (Giles & Musa 2008). Manske et al. (2013) stated that an ER ROM side-to-side difference of less than 5° may predispose a pitcher to injury on account of the fact that increased stresses are exerted on the static glenohumeral stabilisers. The notion of Manske et al. (2013) supported the absence of ER ROM side-to-side differences amongst the uninjured group, but not amongst the cricketers in our study who did in fact sustain injuries during the season, but who also did not show any side-to-side differences in ER ROM.

In the uninjured group, TA ROM (IR plus ER ROM) was less on the dominant side than on the non-dominant side, whilst in the injured group, similar TA ranges were found. It appears that our results are different in this area, as no difference was found in TA ROM in elite fast bowlers (Stuelcken et al. 2008) or cricketers (Giles & Musa 2008). The cricketers who participated in the study by Giles and Musa (2008) were injury-free at the time of testing, although no subgroup analysis was shown, whilst the fast bowlers in the study by Stuelcken et al. (2008) showed symmetries in TA ROM for both groups, the one experiencing pain and the other experiencing no pain.

However, the median dominant TA ROM was less than 180°, which is not considered desirable (Manske et al. 2013). In order to achieve the desired ROM during throwing, compensations from other segments in the kinetic chain are made, including the scapulothoracic and spinal segments (Manske et al. 2013). Our findings amongst the injured cricketers correspond to the suggestion of Manske et al. (2013) that anatomical GIRD, which is normal in overhead-performing athletes, occurs when there is less than an 18°– 20° loss of IR, but when TA ROM is symmetrical. However, the GIRD value amongst our uninjured group was normal (less than 18°–20°), but the TA ROM side-to-side difference was more than 5°, which is predictive of future shoulder injury (Manske et al. 2013). Nonetheless, the group mean results may be different from those of each cricketer inspected on an individual basis.

This is the first study investigating the relationship between the pectoralis minor muscle length and shoulder pain in cricketers. The pectoralis minor muscle length and its predisposing role in injury seem to differ in other studies. Swimmers with shoulder pain have shortened pectoralis minor muscles (Tate et al. 2012), whilst Borstad and Ludewig (2005) found that pain-free volunteers with short pectoralis minor muscles exhibited kinematics similar to those associated with subacromial impingement. They found that in pain-free individuals, a shorter pectoralis minor muscle is associated with the anterior tipping of the scapula at higher levels of arm elevation and more scapula IR at lower levels of arm elevation (Borstad & Ludewig 2005).

However, in our study, it appears that the shortening of pectoralis minor may have acted in a protective capacity, because of the fact that this asymmetry was only present in the uninjured group.

The preventative role of these asymmetries might not be confined only to the glenohumeral joint. Similar asymmetries have been found lower down in the kinetic chain – such as asymmetrical core muscle thickness in uninjured adolescent fast bowlers (Gray et al. 2016; Martin, Olivier & Benjamin 2017; Olivier, Stewart & Mckinon 2013). These abdominal adaptations may be linked to changes further up the kinetic chain, resulting in asymmetrical upper limb muscle changes, as are evident on the dominant upper half of the chest, the dominant upper arm and the forearm in fast bowlers (Grobbelaar 2003). These asymmetries, found in multiple areas of the kinetic chain, may be because of the nature of cricket, which involves asymmetrical limb movements during batting, bowling and fielding. Throwing whilst fielding is a major source of injury in cricket, whilst fielding is a requirement of all cricketers irrespective of their batting or bowling speciality (Robert, Callaghan & Jeffriess 2014). Fielders require great strength to retrieve and throw the ball over long distances to dismiss batsmen (Robert et al. 2014) and to resist distraction forces applied to the glenohumeral joint during the deceleration phase of throwing (Dashottar & Borstad 2012; Escamilla & Andrews 2009). The repeated actions may develop the upper arm to handle the specific functional requirement in order to protect the structures against injury, which seems to play a more important role than the fact that the pectoralis minor muscle is shortened on the dominant side of the uninjured players.

### Strengths and limitations

The strength of our study lies in the fact that it was a longitudinal prospective study, as most studies in this field employ cross-sectional or retrospective study designs. However, the nature of our study does not allow for causality to be determined and only mere associations could be drawn. The incorporation of a regression analysis in a future study with a larger sample size can be used to determine predictors of injury whilst considering a variety of determinants. Differentiation between shoulder pain and asymmetries related to batting, bowling, fielding and wicket-keeping falls outside the realm of our study because a larger sample size is needed to do such subgroup analyses. Confounding factors such as differences in the number and extent of previous injuries, training, leisure activities, diet and lifestyle play a role in the predisposition to injury and were not accounted for in our study.

## Conclusion

Pre-season shoulder pain, which does not limit a cricketer from participating in cricket-related activities, may be a precursor to time-loss shoulder injuries during the season in cricketers. The presence of pain should be investigated during the pre-season screening phase for preventative programmes so that shoulder injuries can be put in place early on. With the exception of ER ROM, side-to-side differences in terms of all of the independent variables (IR ROM, TA ROM and pectoralis minor muscle length distance) were found to be more pronounced amongst the uninjured players.
